# Pancreatic Intraductal Papillary Mucinous Neoplasm in a Carrier of a Reported Pathogenic BRCA2 Variant: Diagnostic Considerations and Management Using European and International Guidelines

**DOI:** 10.7759/cureus.116881

**Published:** 2026-09-24

**Authors:** Julio C Balderas-Ortega, Yoeli Escandon-Espinoza, Katia Lopez Garcia, Gabriela Jasso Guerrero

**Affiliations:** 1 Gastrointestinal Endoscopy, Hospital Regional de Alta Especialidad Bicentenario de la Independencia, Instituto de Seguridad y Servicios Sociales de los Trabajadores del Estado (ISSSTE), Tultitlán, MEX; 2 Pathology, Hospital Regional de Alta Especialidad Bicentenario de la Independencia, Instituto de Seguridad y Servicios Sociales de los Trabajadores del Estado (ISSSTE), Tultitlán, MEX

**Keywords:** brca2, endoscopic ultrasound, european guidelines, eus-fna pancreatic lesions, intraductal papillary mucinous neoplasm, pancreatic cyst, pancreatic surveillance, risk of pancreatic cancer

## Abstract

Pathogenic germline variants in BRCA2 are associated with increased susceptibility to pancreatic ductal adenocarcinoma and may prompt pancreatic surveillance in selected high-risk individuals. Whether BRCA2 status increases the prevalence or alters the biological behavior of intraductal papillary mucinous neoplasms (IPMNs) remains uncertain. We report a 71-year-old Latin American woman with a reported heterozygous pathogenic BRCA2 variant and a remote history of stage IA hormone receptor-positive, HER2-negative breast carcinoma. The original molecular genetics report was unavailable; therefore, the exact variant designation, testing platform, laboratory classification, and American College of Medical Genetics and Genomics (ACMG)/Association of Molecular Pathology (AMP) evidence could not be independently verified.

In June 2025, during long-term oncologic follow-up, positron emission tomography-computed tomography incidentally identified a 21×21 mm pancreatic tail cyst. Magnetic resonance cholangiopancreatography (MRCP) in July 2025 demonstrated a 22×20 mm cyst communicating with the main pancreatic duct (MPD), which measured 5 mm. Endoscopic ultrasound (EUS) demonstrated a 20×23 mm septated cyst without mural nodules or a solid component. Serum carbohydrate antigen 19-9 (CA 19-9) was 21.4 U/mL. Cyst fluid glucose was 12 mg/dL, amylase 316 U/L, and carcinoembryonic antigen (CEA) 247 ng/mL. Cytologic examination and an adequate cell-block specimen obtained by EUS-guided fine-needle aspiration (EUS-FNA) demonstrated papillary mucinous epithelium with gastric-type features, favoring a low-grade IPMN. No immunohistochemical studies were performed, and the specimens were evaluated by a gastrointestinal pathologist.

The case was reviewed by a multidisciplinary team in August 2025. The 5 mm MPD represented a relative indication for surgery under the 2018 European evidence-based guidelines and a worrisome feature under contemporary international criteria, but no absolute or high-risk indication for resection was identified. Surveillance was selected. Computed tomography (CT) in December 2025 showed a stable 22×20 mm cyst and a 5 mm MPD; serum CA 19-9 was 24.7 U/mL. In May 2026, the cyst measured 22×21 mm, and the MPD remained 5 mm. The patient remained clinically stable and asymptomatic without pancreatitis, obstructive jaundice, abdominal pain, weight loss, or other symptoms attributable to the lesion. This case illustrates the integration of lesion-specific morphology, ductal anatomy, pathology, longitudinal imaging, and hereditary-risk context without using reported BRCA2 status alone as an indication for pancreatic resection.

## Introduction

Pathogenic germline variants in BRCA2 impair homologous recombination-mediated DNA repair and are associated with an increased risk of pancreatic ductal adenocarcinoma [1-4]. This association provides part of the rationale for pancreatic surveillance using magnetic resonance imaging (MRI)/magnetic resonance cholangiopancreatography (MRCP) and endoscopic ultrasound (EUS) in selected high-risk individuals [4,5].

Intraductal papillary mucinous neoplasms (IPMNs) are mucin-producing pancreatic precursor lesions with heterogeneous malignant potential. The 2018 European evidence-based guidelines classify findings as absolute or relative indications for resection [6]. Absolute indications include tumor-related jaundice, a contrast-enhancing mural nodule of at least 5 mm or a solid mass, positive cytology for high-grade dysplasia or malignancy, and main pancreatic duct (MPD) dilation of at least 10 mm. Relative indications include MPD dilation of 5-9.9 mm, cyst growth of at least 5 mm/year, serum carbohydrate antigen 19-9 (CA 19-9) above 37 U/mL in the absence of jaundice, cyst diameter of at least 40 mm, new-onset diabetes mellitus, IPMN-related acute pancreatitis, and an enhancing mural nodule smaller than 5 mm [6,7]. The 2024 International Evidence-Based Kyoto Guidelines provide a contemporary framework that revises high-risk stigmata and worrisome features and incorporates EUS findings and EUS-guided fine-needle aspiration (EUS-FNA) cytology when performed [8].

Whether BRCA2 variants increase IPMN prevalence or confer a distinct biological phenotype remains uncertain. Available studies have produced contrasting findings [9-11]. This report describes a patient with a reported pathogenic BRCA2 variant and a pancreatic mucinous cystic lesion with ductal communication and cytologic/cell-block findings favoring a low-grade, gastric-type IPMN. The management decision and longitudinal outcome are examined using European and contemporary international risk-stratification principles.

## Case presentation

A 71-year-old woman of Latin American origin, evaluated at a tertiary care hospital of the Instituto de Seguridad y Servicios Sociales de los Trabajadores del Estado (ISSSTE) in Tultitlán, State of Mexico, Mexico, had a history of hypertension, type 2 diabetes mellitus, and left breast carcinoma. Mammography had identified a left breast nodule, and biopsy demonstrated invasive carcinoma with lymphovascular invasion, estrogen receptor expression of 100%, progesterone receptor expression of 40%, and negative human epidermal growth factor receptor 2 status. She underwent breast-conserving surgery with axillary evaluation; all 15 lymph nodes were negative. The tumor was classified as stage IA (T1bN0M0). Adjuvant treatment consisted of hypofractionated radiotherapy (42.56 Gy in 16 fractions plus a 10 Gy boost) and anastrozole.

Genetic testing documented a reported heterozygous pathogenic variant in the BRCA2 gene, located on chromosome 13q13.1. However, the original molecular genetics report was unavailable for review. Therefore, the Human Genome Variation Society (HGVS) designation, rs identifier, exon/intron location, protein-level consequence, variant-level genomic coordinates, testing platform, performing laboratory, laboratory classification, and American College of Medical Genetics and Genomics (ACMG)/Association of Molecular Pathology (AMP) evidence supporting pathogenicity could not be independently verified. Accordingly, the finding is referred to throughout this report as a "reported pathogenic BRCA2 variant", and no variant-specific genotype-phenotype inference is made.

In June 2025, during long-term oncologic follow-up, positron emission tomography (PET)-computed tomography (CT) showed no evidence of recurrent breast cancer. Two right upper lobe pulmonary nodules measuring 5.4×5.1 mm and 3.2×3.2 mm showed no increased metabolic activity. The same study incidentally identified a 21×21 mm cystic lesion in the pancreatic tail (Figure 1).

**Figure 1:**
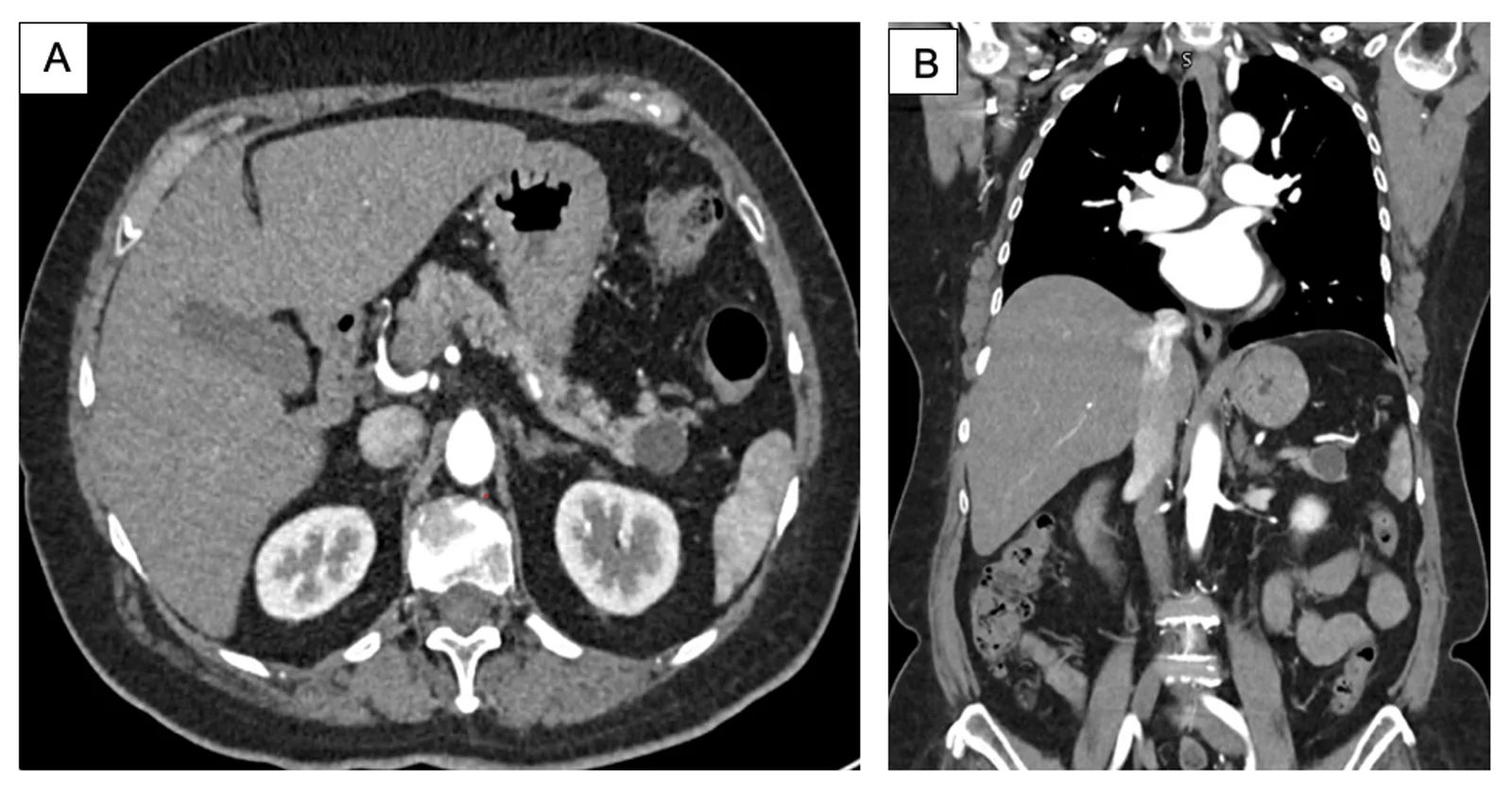
Computed tomography findings of the pancreas. (A) Axial contrast-enhanced computed tomography image demonstrating an incidental cystic lesion in the tail of the pancreas. (B) Coronal contrast-enhanced computed tomography image confirming the pancreatic tail cystic lesion. No solid component or enhancing mural nodule is visible on the displayed images.

MRCP performed in July 2025 demonstrated a 22×20 mm pancreatic tail cyst communicating with the MPD. The MPD measured 5 mm. This documented ductal communication supported IPMN within the differential diagnosis.

EUS demonstrated homogeneous pancreatic parenchyma in the head and body. In the tail, a round 20×23 mm cystic lesion contained a thin internal septum. No mural nodule or solid component was identified, and the interface with adjacent splenic vessels was preserved. The MPD measured 5 mm. The lesion was soft on qualitative elastography. Because cyst characterization could influence management, EUS-FNA was performed using a 19-gauge needle (Figure 2).

**Figure 2:**
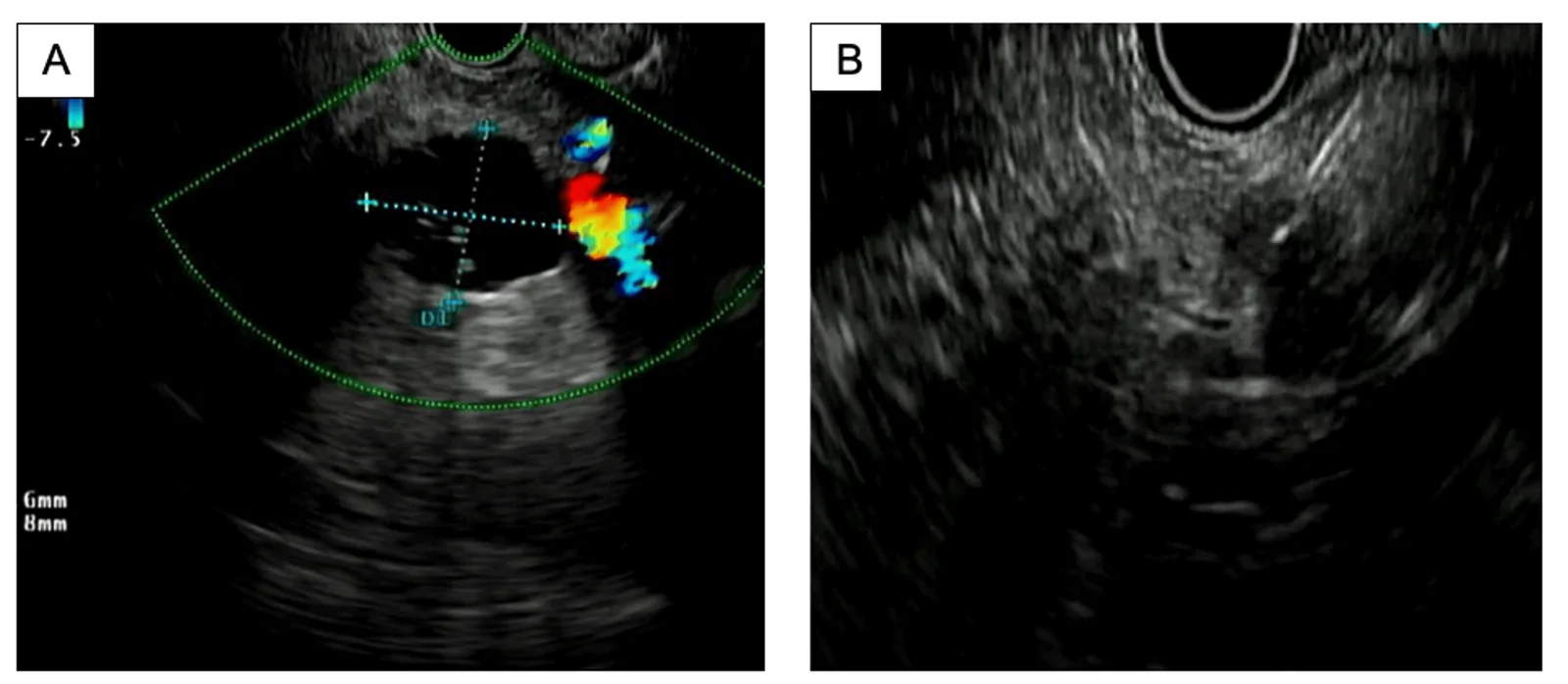
Endoscopic ultrasound findings of the pancreas. (A) Endoscopic ultrasound image with color Doppler demonstrating a 20×23 mm septated cystic lesion in the tail of the pancreas. (B) Endoscopic ultrasound-guided fine-needle aspiration of the pancreatic cystic lesion, with the aspiration needle visualized within the lesion.

Serum CA 19-9 was 21.4 U/mL. Cyst fluid analysis showed glucose of 12 mg/dL, amylase of 316 U/L, and carcinoembryonic antigen (CEA) of 247 ng/mL. The low cyst fluid glucose and elevated CEA supported a mucinous cystic lesion but were not considered sufficient to distinguish IPMN from mucinous cystic neoplasm (MCN) or determine the grade of dysplasia [12-14].

Cytologic examination and cell-block preparation from the EUS-FNA specimen were considered adequate for diagnostic evaluation. The specimens demonstrated papillary mucinous epithelium with gastric-type features, favoring a low-grade IPMN (Figure 3).

**Figure 3:**
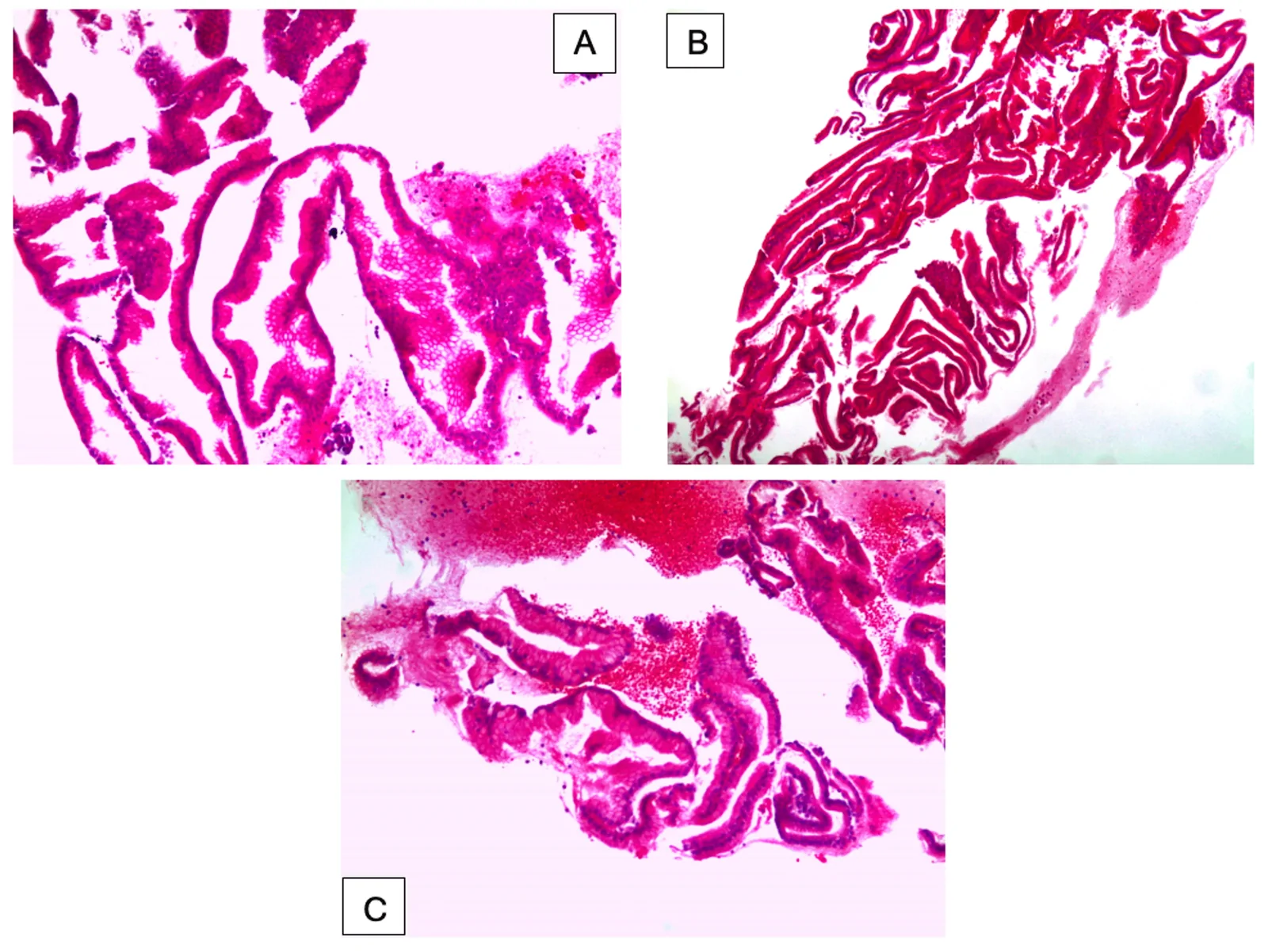
Cytologic and cell-block findings of the pancreatic cystic lesion (hematoxylin and eosin stain). (A) Cytologic specimen obtained by EUS-FNA demonstrating papillary mucinous epithelial fragments with features favoring a low-grade, gastric-type IPMN (×40). (B) Cell-block section prepared from the EUS-FNA specimen showing papillary mucinous epithelium with gastric-type features, favoring low-grade IPMN (×20). (C) Additional cell-block section demonstrating papillary mucinous epithelial architecture (×20). The specimen was considered adequate for diagnostic evaluation and was interpreted by a gastrointestinal pathologist. No immunohistochemical studies were performed. EUS-FNA: endoscopic ultrasound-guided fine-needle aspiration; IPMN: intraductal papillary mucinous neoplasm

Immunohistochemical studies were not performed. The material was evaluated by a gastrointestinal pathologist, and no independent second pathology review was performed.

The case was reviewed by a multidisciplinary team in August 2025. Under the European evidence-based guidelines, the patient had no absolute indication for resection: there was no obstructive jaundice, enhancing mural nodule of at least 5 mm, cytology positive for high-grade dysplasia or carcinoma, or MPD diameter of at least 10 mm [6]. The MPD diameter of exactly 5 mm met one relative criterion. The cyst measured less than 40 mm, serum CA 19-9 was below 37 U/mL, there was no IPMN-related pancreatitis, and the patient's diabetes preceded detection of the pancreatic lesion [6,7]. Considering the lesion-specific findings, age, comorbidities, available pathology, hereditary-risk context, and absence of an absolute surgical indication, surveillance rather than immediate distal pancreatectomy was selected.

Longitudinal imaging demonstrated no clinically meaningful morphologic progression. In December 2025, CT showed a 22×20 mm pancreatic tail cyst and an unchanged MPD diameter of 5 mm. Serum CA 19-9 was 24.7 U/mL, remaining below the guideline threshold of 37 U/mL. Follow-up CT in May 2026 demonstrated a cyst measuring 22×21 mm, while the MPD remained 5 mm. The 1-mm dimensional difference was not interpreted as evidence of clinically meaningful interval growth.

At the most recent follow-up, the patient remained clinically stable and asymptomatic, without episodes of acute pancreatitis, obstructive jaundice, abdominal pain, weight loss, or other symptoms attributable to the pancreatic lesion.

## Discussion

BRCA2, pancreatic cancer risk, and pancreatic cysts

BRCA2 participates in homologous recombination-mediated DNA repair through its interaction with RAD51 [1,2]. Pathogenic germline BRCA2 variants are established susceptibility factors for pancreatic ductal adenocarcinoma and contribute to the rationale for surveillance in selected high-risk individuals [3-5,15]. In this patient, however, the original molecular report was unavailable; therefore, the reported pathogenic classification could not be independently reassessed. More importantly, an association between BRCA2 and pancreatic cancer should not be interpreted as evidence that an individual pancreatic cyst is caused by the germline alteration.

Evidence concerning pancreatic cyst and IPMN prevalence among germline-mutation carriers is inconsistent. Cao et al. found no significant difference in pancreatic cyst prevalence between BRCA-positive, BRCA-negative, and untested individuals (3.6%, 2.6%, and 3.2%, respectively; p=0.64), and high-risk cyst characteristics were similar among groups [10]. In contrast, Roch et al. reported IPMN in 18% of germline-mutation carriers in a prospective pancreatic surveillance population and found germline-mutation status to be independently associated with IPMN (OR: 3.2; p<0.01); prevalence varied by mutation type and was 24% among BRCA2 carriers [11]. Earlier work by Roch et al. also addressed pancreatic surveillance in BRCA1/2 mutation carriers [9]. These contrasting observations support continued investigation but do not establish that BRCA2 directly causes IPMN or defines a distinct BRCA2-associated IPMN phenotype.

Diagnostic classification and differential diagnosis with MCN

The low cyst fluid glucose concentration and elevated CEA supported classification as a mucinous pancreatic cystic neoplasm but cannot independently distinguish IPMN from MCN or establish dysplasia grade [12-14]. These biomarkers must therefore be interpreted in conjunction with cyst morphology, ductal anatomy, and pathologic findings.

MCN deserves specific consideration because the lesion occurred in the pancreatic tail of a woman. In this case, however, MRCP demonstrated communication between the cyst and the MPD, favoring IPMN. In addition, adequate EUS-FNA cytology and cell-block material demonstrated papillary mucinous epithelium with gastric-type features, favoring low-grade IPMN. Nevertheless, the diagnosis was not established from a surgically resected specimen. Definitive assessment of features such as ovarian-type stroma, which would support MCN, was therefore unavailable. The diagnosis should consequently be interpreted as a favored clinicopathologic diagnosis of IPMN rather than a surgically confirmed diagnosis.

European and contemporary international risk stratification

The 2018 European evidence-based guidelines classify an MPD diameter of 5-9.9 mm as a relative indication for surgery, whereas absolute indications include higher-risk findings such as obstructive jaundice, an enhancing mural nodule of at least 5 mm or solid mass, high-grade or malignant cytology, and MPD dilation of at least 10 mm [6]. In this patient, the MPD measured exactly 5 mm, placing her at the lower boundary of the relative-indication range. The lesion remained smaller than 40 mm, no enhancing mural nodule or solid component was detected, serum CA 19-9 remained below 37 U/mL, and the available cytologic/cell-block material favored low-grade disease.

The 2024 International Evidence-Based Kyoto Guidelines revise high-risk stigmata and worrisome features and incorporate EUS findings and EUS-FNA cytology when performed [8]. Under this contemporary framework, MPD dilation of 5-9.9 mm is a worrisome feature rather than, by itself, a mandatory indication for surgery. Thus, both frameworks support integrating duct diameter with the overall clinical and morphologic profile rather than treating a 5 mm MPD measurement as an isolated indication for resection.

The longitudinal findings add clinically relevant information. From June 2025 through May 2026, cyst dimensions remained approximately 21-22 mm, and the MPD remained 5 mm. Serum CA 19-9 remained below 37 U/mL, and no pancreatitis, jaundice, or other lesion-attributable symptoms developed. These observations document short-term stability but do not eliminate future malignant potential or justify discontinuation of surveillance.

Role and limitations of EUS-guided cyst fluid analysis

EUS provides detailed assessment of mural nodules, septations, cyst wall characteristics, vascular relationships, and ductal anatomy. EUS-guided sampling is most useful when its results are expected to influence management [16]. Cyst fluid CEA and glucose can support the classification of a cyst as mucinous, but neither reliably determines dysplasia grade [12-14]. In the present case, biomarker findings, documented ductal communication, and mucinous epithelium were concordant with a mucinous pancreatic neoplasm favoring IPMN.

The FNA specimen and cell block were adequate for diagnostic evaluation and were interpreted by a gastrointestinal pathologist. However, no immunohistochemical studies or independent second pathology review were performed. Moreover, EUS-FNA evaluates only part of a cystic lesion and therefore cannot completely exclude unsampled areas of high-grade dysplasia or invasive carcinoma [16].

Role of the reported BRCA2 status in management

Neither the 2018 European IPMN criteria nor contemporary international IPMN risk-stratification principles establish BRCA2 status alone as an indication for pancreatic resection [6,8]. The reported BRCA2 finding provides hereditary-risk context and supports careful pancreatic assessment but should not replace lesion-specific morphologic, clinical, and pathologic risk stratification. Accordingly, no lower surgical threshold is proposed in this report on the basis of BRCA2 status.

Advanced EUS modalities, including needle-based confocal laser endomicroscopy and through-the-needle biopsy, may improve characterization in selected pancreatic cystic lesions, but their incremental diagnostic yield, adverse events, availability, and cost should be considered [17-19]. These techniques complement rather than replace established clinical and morphologic criteria.

Molecular imaging is an evolving field for diagnosis, prognosis, and treatment assessment in gastrointestinal malignancies, including pancreatic cancer [20]. Its relevance to the present case is contextual: current molecular imaging approaches do not replace established morphologic, endoscopic, cytologic, or histopathologic criteria for IPMN risk stratification.

Limitations

This report has several limitations. First, a single case cannot establish a causal relationship between the reported BRCA2 variant and the pancreatic lesion or define a BRCA2-specific IPMN phenotype. Second, the original molecular genetics report was unavailable; consequently, the exact HGVS designation, testing platform, performing laboratory, laboratory classification, and ACMG/AMP evidence could not be independently verified.

Third, diagnostic misclassification remains possible. Although MRCP demonstrated ductal communication and adequate EUS-FNA cytology and cell-block material interpreted by a gastrointestinal pathologist favored a low-grade, gastric-type IPMN, no surgical specimen was available for definitive classification or assessment for ovarian-type stroma. No immunohistochemical studies or independent second pathology review were performed.

Fourth, EUS-FNA samples only part of the lesion and cannot exclude unsampled high-grade dysplasia or invasive carcinoma. Fifth, measurement variability must be considered because the MPD diameter was exactly 5 mm, at a guideline-defined threshold, and small differences in cyst dimensions across imaging studies and modalities may reflect technical rather than biological change. Sixth, detection bias is possible because the lesion was incidentally identified during oncologic surveillance.

Finally, although longitudinal imaging through May 2026 demonstrated no clinically meaningful morphologic progression and the patient remained asymptomatic, the follow-up period remains limited. The observed short-term stability cannot establish long-term biological behavior, exclude subsequent progression, or support inference of a BRCA2-specific phenotype.

## Conclusions

A pancreatic mucinous cystic lesion detected in a carrier of a reported pathogenic BRCA2 variant should be evaluated according to lesion-specific clinical, morphologic, ductal, and pathologic features rather than attributed to hereditary status alone. In this patient, MRCP demonstrated communication of the pancreatic tail cyst with the MPD, and adequate EUS-FNA cytology and cell-block material favored a low-grade, gastric-type IPMN. MCN remained an appropriate differential consideration, but documented ductal communication favored IPMN.

The MPD diameter of 5 mm represented a relative indication for surgery under the 2018 European evidence-based guidelines and a worrisome feature under contemporary international risk stratification rather than an isolated indication for resection. Multidisciplinary surveillance was selected. During follow-up through May 2026, the cyst remained approximately 22 mm, the MPD remained 5 mm, serum CA 19-9 remained below 37 U/mL, and the patient remained asymptomatic without pancreatitis or obstructive jaundice.

These longitudinal observations support the continuation of structured surveillance but do not establish benign behavior. Current evidence remains insufficient to conclude that BRCA2 carrier status alone produces a distinct IPMN phenotype or warrants a separate surgical threshold.
